# Emergency administration of fibrinogen concentrate for hemorrhage: A protocol for systematic review and meta-analysis

**DOI:** 10.1097/MD.0000000000025099

**Published:** 2021-03-12

**Authors:** Yuki Itagaki, Mineji Hayakawa, Yuki Takahashi, Kazuma Yamakawa

**Affiliations:** a Emergency and Critical Care Centre, Sapporo City General Hospital; b Department of Emergency Medicine, Hokkaido University Hospital, Sapporo, Hokkaido; c Department of Emergency Medicine, Osaka Medical College, Osaka, Japan.

**Keywords:** coagulopathy, fibrinogen concentrate, hemorrhage, transfusion

## Abstract

**Introduction:**

The occurrence of massive hemorrhages in various emergency situations increases the need for blood transfusions and the risk of mortality. Use of fibrinogen concentrate (FC) may increase plasma fibrinogen levels more rapidly than the use of fresh-frozen product or cryoprecipitate. However, thus far, the efficacy of FC in significantly improving the risk of mortality and significantly reducing transfusion requirements has not been effectively demonstrated in several systematic reviews and meta-analyses.

**Methods and analysis:**

We will conduct a systematic review and meta-analysis of FC for hemorrhages in emergency situations. We will include controlled trials, but will exclude randomized controlled trials in elective surgeries. We will include patients with hemorrhages in emergency situations. Intervention will be emergency supplementation of FC. The control group will be administered with ordinal transfusion or placebo. The primary outcome of the study is in-hospital mortality.

We will search in electronic databases such as MEDLINE (PubMed), Web of Science, and the Cochrane Central Register of Controlled Trials. Two reviewers will independently screen the title and abstract, retrieve the full text of the selected articles, and extract the essential data. We will apply uniform criteria for evaluating the risk of bias associated with individual randomized controlled trial based on the Cochrane risk of bias tool. Values of the risk ratio will be expressed as a point estimate with 95% confidence intervals (CIs). Data of continuous variables will be expressed as the mean difference along with their 95% CIs and *P* values. We will assess the strength of evidence using the Grading of Recommendations Assessment, Development and Evaluation approach.

**Ethics and dissemination:**

This systematic review will provide physicians with updated information on the efficacy and safety of using FC for hemorrhage in emergency settings. Approval from the ethics board and patient consent were not required in our study.

This study protocol has been funded through a protocol registry. The registry number is UMIN000041598.

## 1 Introduction

The occurrence of massive hemorrhages in various emergency situations, such as severe trauma, major surgeries, and postpartum, increases the need for blood transfusions and the risk of mortality.^[1–3]^ Fibrinogen levels deteriorate faster than other coagulation factors in conditions such as severe trauma, surgical bleeding, and postpartum.^[4–7]^ Thus, the aggressive complementation of fibrinogen plays an important role in hemostasis.^[8,9]^

Fibrinogen can be supplemented with fresh-frozen plasma (FFP), cryoprecipitate, or fibrinogen concentrate (FC). FFP and cryoprecipitate must be thawed before use, which is a time-consuming process,^[10]^ and ABO compatibility must be usually confirmed before their administration.^[11]^ However, FC does not require a thawing process and confirmation of ABO compatibility.^[11]^ Furthermore, FC may increase plasma fibrinogen levels much more rapidly than FFP or cryoprecipitate^[12]^ and reduce transfusion volume and immunogenic or development of infectious complications.^[13]^

Although several systematic reviews and meta-analyses on randomized controlled trials (RCTs) of major surgery have been published,^[14–17]^ the majority of these reviews included only elective situations and not emergency settings. An advantage of FC is that it can rapidly supplement the massive amount of fibrinogen.^[18]^ Therefore, although the advantage of FC will be clarified in emergency settings, these RCTs may not reveal the advantage of FC. Furthermore, although there have been trauma-related RCTs published recently, their results have not shown the efficacy of FC in improving the survival ratio.^[12,19–21]^. In addition, Stabler et al. published a meta-analysis of these RCTs in 2020 indicating that FC does not show any significant improvement in mortality or any significant reduction in transfusion requirements.^[22]^

Few systematic reviews and meta-analyses have investigated the beneficial effect of FC. Thus, the efficacy of FC massive bleeding in these situations remains ambiguous. Therefore, we aimed to conduct a new systematic review and meta-analysis of studies on the safety and efficacy FC in emergency situations, compared with those of established transfusion strategies.

## 2 Methods and analysis

### 2.1 Protocol registration

This study protocol has been registered in the UMIN (UMIN registration number: UMIN0000415989, URL https://upload.umin.ac.jp/cgi-open-bin/ctr_e/ctr_view.cgi?recptno=R000047487). This systematic review and meta-analysis will be reported according to The Preferred Reporting Items for Systematic Reviews and Meta-Analyses Protocols guidelines.^[23]^

### 2.2 Database search

To retrieve relevant articles, we will perform a literature search in the following major electronic bibliographic databases: MEDLINE (PubMed), Web of Science, and the Cochrane Central Register of Controlled Trials. We will examine the references cited in relevant articles to ascertain whether additional studies can be found. The details of the search strategy are available in Supplemental content (see Appendixes 1, 2, and 3).

### 2.3 Types of studies

We will include controlled trials (including RCTs and other controlled trials) published until October 17, 2020. Studies will be excluded if they do not report the population, treatment, or outcomes of interest clearly. We will also exclude RCTs on elective surgeries to assess the effect of FC on uncontrolled bleeding in emergency situations. Animal studies will also be excluded. Gray literature such as conference proceedings and abstracts will be included. If 2 or more studies were published using the same or overlapping cohorts, then the most recent or larger cohort will be included. No language restrictions will be applied. For non-English language publications, appropriate translation services will be utilized.

### 2.4 Study population

We will include patients admitted to the hospital with emergency hemorrhage. The causes of hemorrhages that will be included are trauma, postpartum, cardiovascular diseases, and emergency surgery. We will exclude elective surgery patients. We will not restrict our review by country.

### 2.5 Intervention and control

Intervention types of interest will be the emergency supplementation of fibrinogen in the treatment for hemostasis. The control groups will be administered ordinal transfusion treatments (i.e., FFP) or placebo. We will not restrict our review by the product type, the amount, and the timing of administration of FC.

### 2.6 Outcomes

The primary outcome will be in-hospital mortality due to all causes. The secondary outcomes will be the quantity of the transfused blood within 24 hours, blood loss within 24 hours, thrombotic events (i.e., deep venous thromboses, pulmonary embolization, myocardial infarctions, strokes), multiple organ failures, length of intensive care unit (ICU) stays, and length of hospital stays.

### 2.7 Selection of studies

Citations will be stored, and duplicates will be removed using EndNote Software (Thomson Reuters, Toronto, Ontario, Canada). We will use Rayyan software for the systematic review process.^[24]^ The titles and abstracts of studies retrieved using the search strategy will be screened independently by 2 reviewers (YI and YT) to identify studies that potentially meet the inclusion criteria. The full text of these potentially eligible studies will be retrieved and assessed independently by 2 reviewers (YI and YT). Any disagreement about the eligibility of studies will be resolved through consultation with a third reviewer (KY).

### 2.8 Data extraction

Using a standardized pre-piloted form, data from the included studies will be extracted to assess the quality of the studies and the methods of data synthesis. The extracted information will include data on the following variables: study settings, study populations, baseline characteristics of the participants, details of the interventions and control conditions, study methodologies, outcomes, and assessments of risks of bias. Two independent reviewers (YI and YT) will extract the data independently, and discrepancies will be resolved through discussion with the third author (KY). Missing data will be obtained through request from the authors.

### 2.9 Assessment of risk of bias in individual studies

The 2 independent reviewers (YI and YT) will assess the risk of bias in individual studies as well as the methodological quality of the articles, and disagreements will be resolved by discussion with a third reviewer (KY). We will apply uniform criteria for evaluating the risk of bias associated with individual RCTs based on the tool for assessing risk of bias in randomized trials.^[25]^ Each study will be assessed for

(1) random sequence generation,
(2) allocation concealment,
(3) blinding of participants and personnel,
(4) blinding of related outcome assessment,
(5) incomplete outcome data,
(6) selective reporting, and
(7) other bias.

### 2.10 Data summary

We will perform a meta-analysis when data are available in 1 or more trials according to the “Cochrane Handbook for Systematic Reviews of Interventions.” For binary variables, values for the risk ratio or the odds ratio will be expressed as a point estimate with 95% CIs. Data of continuous variables such as the length of ICU stay will be expressed as their mean difference with 95% CIs and *P* value. If quantitative synthesis is not appropriate for a particular outcome, we will provide a qualitative summary.

### 2.11 Data synthesis

We will provide estimates of the findings from the included studies according to a random-effects model. A random-effects model incorporates statistical heterogeneity and provides a more conservative estimate of the pooled effect size than a fixed-effects mode. We will not perform any multiple imputations for missing data, data synthesis, and analysis of randomized trials.

All statistical analyses including risk of bias within studies and/or across studies will be performed with Review Manager, Version 5.4. (RevMan; The Cochrane Collaboration 2019, The Nordic Cochrane Centre, Copenhagen, Denmark).^[26]^ The level of statistical significance will be set at a *P* value of < .05.

### 2.12 Assessment of heterogeneity

Statistical heterogeneity will be assessed using the Mantel-Haenszel *χ*^2^ test and the *I*^2^ (the value of *I*^2^ > 50% = significantly heterogeneity).^[27]^ The presence of clinical heterogeneity will be considered in the decision to conduct a quantitative synthesis of data or to perform sensitivity analyses.

#### 2.12.1 Sensitivity analysis

We will examine the robustness of this meta-analysis by conducting sensitivity analysis according to the different components of the Cochrane risk of bias tool. We will also perform the analysis by excluding studies in which overall risk of bias judgment is high or unclear risk of bias.

#### 2.12.2 Subgroup analysis

Subgroup analyses will be performed according to the type of hemorrhage experienced by the patients (trauma, gastrointestinal hemorrhage, surgery, postpartum), type of surgery (cardiac surgery, other major surgery), country, sample size, published year, timing of administration, and type of FC.

### 2.13 Assessment of reporting bias

To assess publication bias, we will create funnel plots for mortality.

### 2.14 Rating the strength of evidence using the Grading of Recommendations Assessment, Development and Evaluation (GRADE) approach

The 2 authors (YI and YT) will assess the strength of evidence independently, using the GRADE approach.^[28]^ The quality of evidence will be assessed for each outcome and categorized as high, moderate, low, or very low according to the GRADE approach.

### 2.15 Patient and public involvement

Patients and the public were not involved in the design of this protocol.

## 3 Discussion

The use of FC enables the rapid and strong supply of fibrinogen in the serum without the need for confirming ABO compatibility. Thus, FC may change the result of a life-saving procedure compared with an ordinary transfusion strategy consisting of an allogenic transfusion. Thus far, there have been many RCTs that have been performed on the use of FC in elective surgery; however, there is a lack of studies in emergency settings.

Recently, RCTs for the use of FC in trauma or postpartum situations have increased. By summarizing these results, we will update the information on the safety and efficacy of the use of FC that has been previously reported and investigate the effect on newly reported, as mortality rate. This meta-analysis will therefore be one of the first attempts to report the effect of the administration of FC in emergency situations on survival rate.

In this study, as we will focus on emergency bleeding situations such as trauma, gastrointestinal hemorrhage, surgery (regardless of the type), and postpartum, the analysis will summarize multiple aspects. This is the first attempt to conduct a systematic review and meta-analysis on the efficacy and safety of using FC for emergency hemorrhage. Therefore, our findings will provide frontline physicians with updated information on the safety and efficacy of FC in life-saving situations.

## 4 Ethics and dissemination

Approval from an ethics committee was not required since this will be a systematic review and meta-analysis of publicly available data without any direct involvement of human participants. Our findings will be presented at relevant scientific conferences and disseminated through publication in a peer-reviewed journal.

## Acknowledgments

We would like to thank Editage (www.editage.com) for English language editing.

## Author contributions

KY and MH were guarantors and contributed to the conception of the study. The manuscript protocol was drafted by YI and was revised by KY and MH. The search strategy was developed by all of the authors and will be performed by YI and MH. YI and YT will independently extract data from the included studies, assess the risk of bias and complete the data synthesis. KY will arbitrate in cases of disagreement and ensure the absence of errors. All authors approved the publication of this protocol.

**Data curation:** Yuki Itagaki, Yuki Takahashi.

**Formal analysis:** Yuki Itagaki.

**Investigation:** Yuki Itagaki.

**Resources:** Yuki Itagaki.

**Software:** Yuki Itagaki.

**Supervision:** Mineji Hayakawa, Kazuma Yamakawa.

**Writing – original draft:** Yuki Itagaki.

**Writing – review & editing:** Mineji Hayakawa, Kazuma Yamakawa.

## Supplementary Material

Supplemental Digital Content

## Supplementary Material

Supplemental Digital Content

## Supplementary Material

Supplemental Digital Content
