# Supplementary material for: Emergency administration of fibrinogen concentrate for hemorrhage: A protocol for systematic review and meta-analysis
Source: Medicine (Baltimore). 2021 Mar 12;100(10):e25099. doi: 10.1097/MD.0000000000025099 (PMC7969309; doi:10.1097/MD.0000000000025099)
Supplement: Supplemental Digital Content [file medi-100-e25099-s001.pdf]

Appendix 1: CENTRAL search strategy

#1 [mh trauma]
#2 [mh postpartum]

#3 cardiac:ti,ab
#4 cardiovascular:ti,ab
#5 aort*:ti,ab
#6 #3 OR #4 OR #5

#7 [mh "perioperative period"]
#8 perioperative:ti,ab
#9 #7 OR #8

#10 [mh "gastrointestinal hemorrhage"]

#11 Hemorrhage:ti,ab
#12 haemorrhage:ti,ab
#13 bleed:ti,ab
#14 #11 OR #12 OR #13

#15 #1 OR #2 OR #6 OR #9 OR #10 OR #14

#16 [mh fibrinogen]
#17 RiaSTAP:ti,ab
#18 Haemocomplettan:ti,ab
#19 Clottafact:ti,ab
#20 "fibrinogen concentrate":ti,ab
#21 "fibrinogen substitution":ti,ab
#22 #16 OR #17 OR #18 OR #19 OR #20 OR #21

#23 #15 AND #22
