# Supplementary material for: Emergency administration of fibrinogen concentrate for hemorrhage: A protocol for systematic review and meta-analysis
Source: Medicine (Baltimore). 2021 Mar 12;100(10):e25099. doi: 10.1097/MD.0000000000025099 (PMC7969309; doi:10.1097/MD.0000000000025099)
Supplement: Supplemental Digital Content [file medi-100-e25099-s002.pdf]

Appendix 2: MEDLINE(via PubMed) search strategy

#1 trauma[mh]

#2 postpartum[mh]

#3 “cardiac”[tiab]

#4 "cardiovascular"[tiab]

#5 "aort*"[tiab]

#6 #3 OR #4 OR #5

#7 perioperative period[mh]

#8 perioperative[tiab]

#9 #7 OR #8

#10 gastrointestinal hemorrhage[mh]

#11 Hemorrhage[tiab]

#12 haemorrhage[tiab]

#13 bleed[tiab]

#14 #11 OR #12 OR #13

#15 #1 OR #2 OR #6 OR #9 OR #10 OR #14

#16 fibrinogen[mh]

#17 "RiaSTAP"[tiab]

#18 "Haemocomplettan"[tiab]

#19 "Clottafact"[tiab]

#20 "fibrinogen concentrate"[tiab]

#21 "fibrinogen substitution"[tiab]

#22 #16 OR #17 OR #18 OR #19 OR #20 OR #21

#23 randomized controlled trial [pt]

#24 controlled clinical trial [pt]

#25 randomized [tiab]

#26 placebo [tiab]

#27 drug therapy [sh]

#28 randomly [tiab]

#29 trial [tiab]
#30 groups [tiab]
#31 #23 OR #24 OR #25 OR #26 OR #27 OR #28 OR #29 OR #30
#32 animals [mh] NOT humans [mh]
#33 #31 NOT #32

#34 #15 AND #22
#35 #34 AND #31
