# Supplementary material for: Emergency administration of fibrinogen concentrate for hemorrhage: A protocol for systematic review and meta-analysis
Source: Medicine (Baltimore). 2021 Mar 12;100(10):e25099. doi: 10.1097/MD.0000000000025099 (PMC7969309; doi:10.1097/MD.0000000000025099)
Supplement: Supplemental Digital Content [file medi-100-e25099-s003.pdf]

Appendix 3: Web of science search strategy

#1 trauma
#2 postpartum

#3 cardiac
#4 cardiovascular
#5 aort*
#6 #3 OR #4 OR #5

#7 “perioperative period”
#8 perioperative
#9 #7 OR #8

#10 "gastrointestinal hemorrhage"

#11 Hemorrhage
#12 haemorrhage
#13 bleed
#14 #11 OR #12 OR #13

#15 #1 OR #2 OR #6 OR #9 OR #10 OR #14

#16 fibrinogen
#17 RiaSTAP
#18 Haemocomplettan
#19 Clottafact
#20 "fibrinogen concentrate"
#21 "fibrinogen substitution"
#22 #16 OR #17 OR #18 OR #19 OR #20 OR #21

#23 #15 AND #22
